# Supplementary material for: Coordinated inflammatory responses dictate Marburg virus control by reservoir bats
Source: Nat Commun. 2024 Feb 28;15:1826. doi: 10.1038/s41467-024-46226-7 (PMC10902335; doi:10.1038/s41467-024-46226-7)
Supplement: Supplementary file 1 — Supplementary Information [file 41467_2024_46226_MOESM1_ESM.pdf]

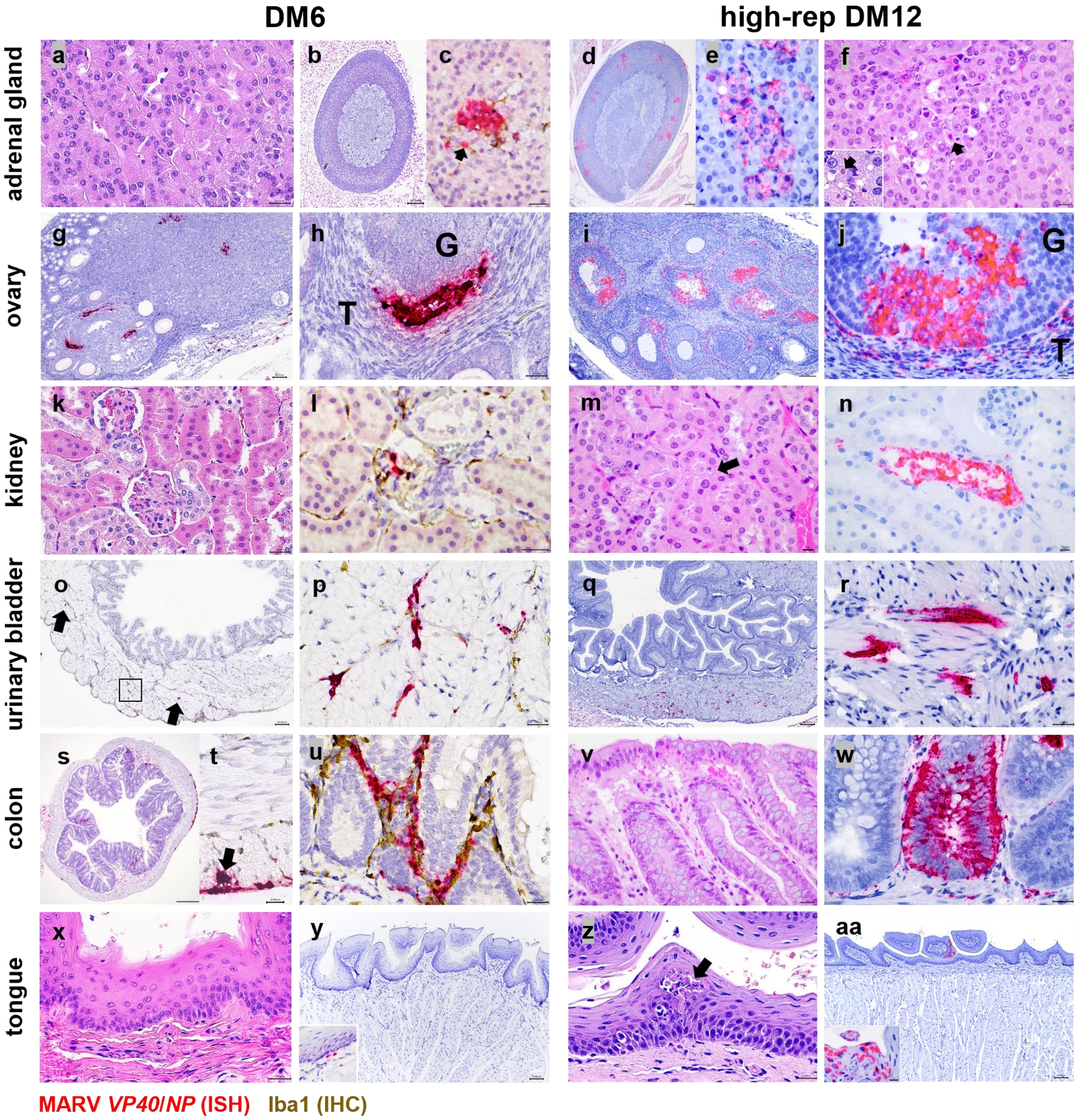

Supplementary Fig. 1 | Histologically-resolvable and Marburg virus disease (MVD)-like viral tissue/cell tropism in IM bats. **a**, H&E staining; *n*=5 bats. Bar (scale): 20µm. **b**, MARV ISH showing lack of MARV replication foci. Bar: 200µm. **c**, Dual MARV ISH (red) and Iba1 IHC (brown). MARV RNA in tissue macrophages (MΦ, arrow) and adrenal cortical cells. Bar: 10µm. **d**, MARV ISH signal in inflammatory foci of high-replicating (high-rep) DM12 bats. Bar: 200µm. **e**, MARV ISH signal in adrenal cortical cells. Bar: 10µm. **f**, H&E. CPE of MARV in adrenal cortical cells include cytoplasmic vacuolation, occasional necrosis (arrow) and rare eosinophilic to amphophilic cytoplasmic inclusions (arrow, inset); *n*=3 bats. Bar: 10µm. **g**, MARV ISH. Bar: 80µm. **h**, MARV ISH. G: granulosa cells, T: theca cells. Bar: 20µm. **i**, MARV ISH. Bar: 100µm. **j**, MARV ISH. Bar: 10µm. **k**, H&E; *n*=5 bats. Bar: 20µm. **l**, Dual ISH-IHC. MARV RNA in Iba1$^+$ glomerular cells. Bar: 10µm. **m**, H&E. CPE of MARV in renal cortical epithelial cells include cytoplasmic vacuolation and occasional pyknosis (arrow); *n*=3 bats. Bar: 10µm. **n**, MARV ISH. Bar: 10µm. **o**, Dual ISH-IHC. Scattered MARV RNA in bladder wall (arrows). Bar: 200µm. **p**, Dual ISH-IHC. Intense MARV RNA within Iba1$^+$ tissue MΦ. Bar: 20µm. **q**, MARV ISH. Bar: 80µm. **r**, Intense MARV ISH signal within smooth muscle cells. Bar: 20µm. **s**, MARV ISH. Bar: 200µm. **t-u**, Dual ISH-IHC. **t**, MARV RNA within Iba1$^+$ tissue MΦ, mesothelial cells and focally within smooth muscle cells (arrow). **u**, Intense MARV signal within Iba1$^+$ lamina propria MΦ. Bars: 20µm. **v**, H&E. Intraepithelial and lamina propria hemorrhage; *n*=1 bat. Bar: 10µm. **w**, MARV ISH. Bar: 20µm. **x**, H&E; *n*=5 bats. Bar: 20µm. **y**, MARV ISH. Bar: 80µm. **z**, H&E. Focus of epithelial cell necrosis (arrow); *n*=1 bat. Bar: 20µm. **aa**, MARV ISH. Bar: 80µm. Inset: MARV RNA in epithelial cells (bar: 10µm). Hematoxylin counterstain (all ISH/dual assays).

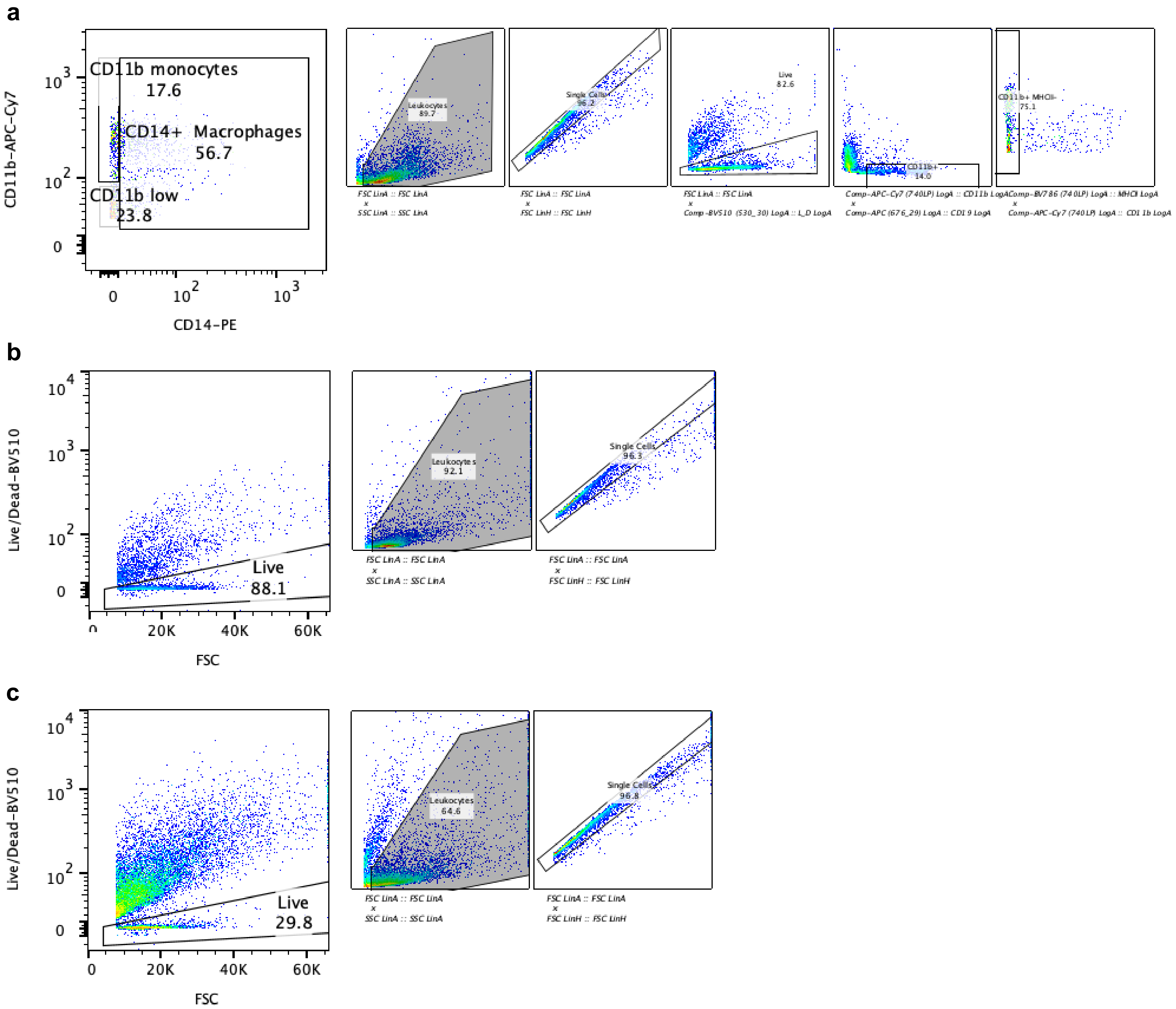

Supplementary Fig. 2 | Flow cytometric analysis and gating ancestry of immune cell populations.
a, Splenocytes from a representative bat were live/dead stained, then gated for leukocytes, single cells, live cells, then specific cell types as indicated. Viable cell proportions were used to calculate the total cells per tissue per animal. **b-c**, Axillary lymph node (ALN) cells from a representative Dex bat (**b**) or Neg bat (**c**) with gating ancestry.

Target mRNA	GenBank Entry Weblink	RNAscope Catalog ID	Probe Design Notes
CD14	https://www.ncbi.nlm.nih.gov/nuccore/XM_016166396.1	861218	20ZZ probe named Rae-CD14 targeting 164-1146 of XM_016166396.1
CD79a	https://www.ncbi.nlm.nih.gov/nuccore/XM_016129923.1	861228	20ZZ probe named Rae-CD79A targeting 2-957 of XM_016129923.1
CD3e	https://www.ncbi.nlm.nih.gov/nuccore/XM_016155454.1	861248	20ZZ probe named Rae-CD3E targeting 109-1067 of XM_016155454.1
TNF	https://www.ncbi.nlm.nih.gov/nuccore/XM_016121699.1	878031-C2	20ZZ probe named Rae-TNF targeting 359-1490 of XM_016121699.1
IL10	https://www.ncbi.nlm.nih.gov/nuccore/XM_016129276.1	878041-C2	20ZZ probe named Rae-IL10 targeting 2-1244 of XM_016129276.1
IL6	https://www.ncbi.nlm.nih.gov/nuccore/XM_016159525.1	878051-C2	20ZZ probe named Rae-IL6 targeting 3060-4182 of XM_016159525.1
**Marburg virus 371bat *NP/VP40***	https://www.ncbi.nlm.nih.gov/nuccore/FJ750958	853531	39ZZ probe named V-MARV-NP-VP40 targeting 3060-4182 of FJ750958.1
**ERB *PPIB***	https://www.ncbi.nlm.nih.gov/nuccore/XM_016141088.1	573731	20ZZ probe named Rae-PPIB targeting 71-974 of XM_016141088.1

Supplementary Table 1. | ERB- or MARV-specific RNA probes used for ISH assays in this study.

Cohort	Neg					Dex					M6					M12				DM6					low-rep DM12		high-rep DM12		
**Bat #**	Bat1	Bat2	Bat3	Bat4	Bat5	Bat1	Bat2	Bat3	Bat4	Bat5	Bat1	Bat2	Bat3	Bat4	Bat5	Bat1	Bat2	Bat3	Bat4	Bat1	Bat2	Bat3	Bat4	Bat5	Bat1	Bat2	Bat3	Bat4	Bat5
Liver	0 (*n*=3)	-	0 (*n*=4)	0 (*n*=3)	0 (*n*=3)	0 (*n*=3)	0 (*n*=3)	0 (*n*=3)	0 (*n*=3)	0 (*n*=3)	1 (*n*=3)	1 (*n*=3)	2 (*n*=3)	1 (*n*=3)	2 (*n*=4)	0 (*n*=3)	0 (*n*=3)	0 (*n*=3)	0 (*n*=3)	3 (*n*=3)	3 (*n*=3)	3 (*n*=3)	3	3 (*n*=4)	0 (*n*=3)	0 (*n*=3)	3 (*n*=4)	3 (*n*=4)	4 (*n*=4)
Spleen	0 (*n*=3)	-	0 (*n*=4)	0 (*n*=3)	0 (*n*=3)	0 (*n*=3)	0 (*n*=3)	0 (*n*=3)	0 (*n*=3)	0 (*n*=3)	2 (*n*=3)	1 (*n*=3)	1 (*n*=2)<br>2 (*n*=1)	0 (*n*=3)	2	1 (*n*=1)/<br>0 (*n*=2)	1 (*n*=2)/<br>0 (*n*=1)	0 (*n*=3)	1 (*n*=1)<br>0 (*n*=2)	3 (*n*=3)	-	4 (*n*=4)	2	3 (*n*=3)	0 (*n*=3)	1 (*n*=2)/<br>0 (*n*=1)	3 (*n*=4)	3 (*n*=4)	3 (*n*=4)
Axillary lymph node	0 (*n*=3)	-	0 (*n*=4)	0 (*n*=3)	-	-	-	0 (*n*=3)	0 (*n*=3)	0 (*n*=3)	2 (*n*=3)	0 (*n*=3)	1 (*n*=3)	0 (*n*=3)	1	0 (*n*=3)	1 (*n*=1)/<br>0 (*n*=2)	0 (*n*=3)	0 (*n*=3)	2 (*n*=2)<br>1 (*n*=1)	-	3 (*n*=4)	2	2 (*n*=3)	1 (*n*=1)/<br>0 (*n*=2)	-	2 (*n*=4)	1 (*n*=4)	2 (*n*=4)
Bone marrow	0 (*n*=3)	-	0	-	-	-	-	-	-	0 (*n*=3)	0 (*n*=3)	-	-	-	0	-	-	-	-	-	-	-	0	0 (*n*=3)	-	-	-	-	-
Mesenteric lymph node	-	-	0	-	-	-	-	-	-	-	-	-	-	-	0	-	-	-	-	-	-	3	-	3	-	-	2	3	2
Lymph node, other	0 (*n*=3)	-	-	-	-	-	-	-	-	-	-	-	-	-	0	-	0	-	-	-	-	3 (*n*=3)	2	1 (*n*=3)	-	-	-	-	3
Skin, inoculation site	-	-	-	-	-	-	-	-	-	-	3 (SC only)	-	-	-	0	-	-	-	-	-	-	-	-	-	-	-	-	2	1
Skin, other - caudal abdomen/perineum	-	-	-	-	-	-	-	-	-	-	0	-	-	-	2	-	-	-	-	-	-	3	-	-	-	-	0	0	1
Skeletal muscle	0 (*n*=3)	-	-	-	-	-	-	-	-	0 (*n*=3)	-	-	-	-	0	-	0	-	-	-	-	0	-	-	-	-	0	-	-
Thymus		-	-	-	-	-	0 (*n*=3)	-	-		-	-	-	-	0 (*n*=4)	-	-	-	-	-	-	0 (*n*=3)	3	2 (*n*=1)<br>1 (*n*=3)	-	-	-	-	-
Aorta	0 (*n*=3)	-	-	-	-	-	0 (*n*=3)	-	-		0 (*n*=3)	-	-	-	0 (*n*=4)	-	0	-	-	-	0 (*n*=3)	0 (*n*=3)	0	0 (*n*=4)	-	-	0	-	0
Heart	0 (*n*=3)	-	0 (*n*=3)	-	-	-	0 (*n*=3)	-	-	0 (*n*=3)	0 (*n*=3)	0 (*n*=3)	-	-	-	-	0	-	-	0 (*n*=3)	0 (*n*=3)	0 (*n*=3)	1	0 (*n*=4)	-	-	1	-	0
Lung	0 (*n*=3)	-	0 (*n*=3)	-	-	-	0 (*n*=3)	-	-	0 (*n*=3)	0 (*n*=3)	-	0 (*n*=3)	0 (*n*=3)	0 (*n*=4)	-	0	-	-	0 (*n*=3)	0 (*n*=3)	-	-	1 (*n*=3)	-	-	1	1	2
Trachea	0 (*n*=3)	-	0	-	-	-	0 (*n*=3)	-	-	-	-	-	-	-	0 (*n*=4)	-	0	-	-	-	-	1 (*n*=3)	0	0 (*n*=3)<br>1 (*n*=1)	-	-	-	0	0
Esophagus	0 (*n*=3)	-	0	-	-	-	0 (*n*=3)	-	-	-	-	-	-	-	0	-	0	-	-	-	-	2 (*n*=3)	0	1 (*n*=3)	-	-	2	0	1
Stomach	-	-	0	-	-	-	-	-	-	-	-	-	-	-	-	-	0	-	-	-	-	0	-	1	-	-	3	3	3
Pancreas	-	-	0	-	-	-	-	-	-	-	-	-	-	-	0	-	0	-	-	-	-	2	-	-	-	-	1	0	1
Small intestine	-	-	0	-	-	-	-	-	-	-	-	-	-	-	0	-	0	-	-	-	-	2	-	2	-	-	3	3	3
Mesentery	-	-	0	-	-	-	-	-	-	-	-	-	-	-	0	-	-	-	-	-	-	2	2	1	-	-	2	2	2
Colon	-	-	0	-	-	-	-	-	-	-	-	-	-	-	0	0	0	-	-	-	-	2	-	2	-	0 (*n*=3)	3	2	3
Colonic lymph node	-	-	0	-	-	-	-	-	-		-	-	-	-	-	0	0	-	-	-	-	-	-	-	-	0 (*n*=3)	2	2 (*n*=3)	-
Kidney	0 (*n*=3)	-	-	-	-	-	0 (*n*=3)	-	-	0 (*n*=3)	0 (*n*=3)	0 (*n*=3)	0 (*n*=3)	0 (*n*=3)	0 (*n*=4)	-	0	-	-	0 (*n*=3)	0 (*n*=3)	0 (*n*=3)	1	1 (*n*=4)	-	-	1	1	1
Urinary bladder	-	-	0	-	-	-	-	-	-	-	0 (*n*=3)	-	-	-	0	-	-	-	-	-	-	-	-	2	-	-	1	2	3
Adrenal gland	0 (*n*=3)	-	-	-	-	-	-	-	-	-	-	0 (*n*=3)	-	-	0 (*n*=4)	-	0	-	-	0 (*n*=1)	0 (*n*=3)	0 (*n*=3)	-	1 (*n*=2)<br>0 (*n*=2)	-	-	3	2	3
Vagina	-	-	-	-	-	-	-	-	-	-	-	-	-	-	0	-	-	-	-	-	-	1 (SM only)	-	-	-	-	-	0	1
Uterus	-	-	-	-	-	-	-	-	-	-	-	-	-	-	0	-	-	-	-	-	-	1	-	-	-	-	-	3	3
Ovary	-	-	-	-	-	-	-	-	-	-	-	-	-	-	-	-	-	-	-	-	-	3	-	-	-	-	-	-	3
Testis	-	-	0	-	-	-	-	-	-	-	-	-	-	-	-	-	-	-	-	-	-	-	-	0	-	-	0	-	-
Penis	-	-	-	-	-	-	-	-	-	-	1 (SM only)	-			-	-	-	-	-	-	-	-	-	-	-	-	1 (ET)	-	-
Brain	-	-	0	-	-	-	-	-	-	-	0	-	-	-	0	-	0	-	-	-	-	-	-	0	-	-	-	0	0
Eye	-	-	-	-	-	-	-	-	-	-	0	-	-	-	-	-	0	-	-	-	-	-	-	-	-	-	0	1	1
Tongue	-	-	0	-	-	-	-	-	-	-	-	-	-	-	0	-	0	-	-	-	-	1 (SM only)	-	-	-	-	2	1	1
Salivary gland	0 (*n*=3)	-	0	-	-	-	-	-	-	0 (*n*=3)	0 (*n*=3)	-	-	-	0	-	0	-	-	-	-	0 (*n*=3)	0	-	-	-	0	0	1 (M-type)

Supplementary Table 2. | MARV ISH scores by tissue and cohort in individual ERBs.

For all ISH assays, bat tissues were scored semi-quantitatively: 0 = MARV not detected; 1 = minimally MARV$^+$ (1-10% of tissue); 2 = occasionally MARV$^+$ (11-25%); 3 = frequently MARV$^+$ (25-75%); 4 = extensively MARV$^+$ (>75%). Assay replicates per bat were *n*=1 unless specified in parentheses. Microscopic features and/or cell types assessed in tissues: liver (hepatocytes, Kupffer cells, MΦ, endothelial cells); spleen (MΦ, follicular dendritic cells [fDCs], ellipsoids/endothelium); axillary lymph node (LN; fDCs, MΦ, capsule); mesenteric LN (MΦ, capsule); LN, other (perirenal, mediastinal, submandibular); skin, inoculation site (epidermis, adipocytes, fibroblasts, MΦ, sebaceous glands); skin, other (epidermis, adipocytes, sebaceous glands); skeletal muscle (pectoral, epaxial); heart (valve, endocardium, interstitium); lung (alveolar septa, circulating mononuclear cells); trachea (epithelium, submucosa, adventitia); esophagus (smooth muscle fibers); stomach (interstitium, epithelium); pancreas (acinar cells); small intestine (interstitium, epithelium, smooth muscle fibers, mesothelium); mesentery (adipocytes, mesothelial cells); colon (interstitium, epithelium, smooth muscle fibers, mesothelium); colonic LN (MΦ, fDCs, capsule); kidney (glomerulus, renal tubules, interstitium); urinary bladder (smooth muscle fibers, interstitium); adrenal gland (cortical cells, interstitium); vagina (smooth muscle fibers, epithelium); uterus (smooth muscle fibers, mesothelium, glandular epithelium); ovary (granulosa/theca cells); penis (epithelium, submucosa); eye (conjunctival epithelium); tongue (epithelium, submucosa); salivary gland (acinar cells). SC = subcutis; SM = submucosa; ET = epithelium; M-type = mucous type acinar cells.
