# Supplementary material for: Coordinated inflammatory responses dictate Marburg virus control by reservoir bats
Source: Nat Commun. 2024 Feb 28;15:1826. doi: 10.1038/s41467-024-46226-7 (PMC10902335; doi:10.1038/s41467-024-46226-7)
Supplement: Supplementary file 3 — Reporting Summary [file 41467_2024_46226_MOESM3_ESM.pdf]

nature portfolio

Corresponding author(s): Jonathan Towner, Joseph Prescott

Last updated by author(s): Feb 7, 2024

Reporting Summary

Nature Portfolio wishes to improve the reproducibility of the work that we publish. This form provides structure for consistency and transparency in reporting. For further information on Nature Portfolio policies, see our Editorial Policies and the Editorial Policy Checklist.

Statistics

For all statistical analyses, confirm that the following items are present in the figure legend, table legend, main text, or Methods section.

n/a	Confirmed	
☐	☒	The exact sample size (*n*) for each experimental group/condition, given as a discrete number and unit of measurement
☐	☒	A statement on whether measurements were taken from distinct samples or whether the same sample was measured repeatedly
☐	☒	The statistical test(s) used AND whether they are one- or two-sided<br>*Only common tests should be described solely by name; describe more complex techniques in the Methods section.*
☐	☒	A description of all covariates tested
☒	☐	A description of any assumptions or corrections, such as tests of normality and adjustment for multiple comparisons
☐	☒	A full description of the statistical parameters including central tendency (e.g. means) or other basic estimates (e.g. regression coefficient) AND variation (e.g. standard deviation) or associated estimates of uncertainty (e.g. confidence intervals)
☐	☒	For null hypothesis testing, the test statistic (e.g. *F*, *t*, *r*) with confidence intervals, effect sizes, degrees of freedom and *P* value noted<br>*Give P values as exact values whenever suitable.*
☒	☐	For Bayesian analysis, information on the choice of priors and Markov chain Monte Carlo settings
☒	☐	For hierarchical and complex designs, identification of the appropriate level for tests and full reporting of outcomes
☒	☐	Estimates of effect sizes (e.g. Cohen's *d*, Pearson's *r*), indicating how they were calculated

Our web collection on statistics for biologists contains articles on many of the points above.

Software and code

Policy information about availability of computer code

Data collection	Abaxis VetScan HM5 v2.4, Applied Biosystems 7500 software v2.0.6, Abaxis Piccolo Xpress Chemistry Analyzer software v3.1.37, Stratedigm CellCapTure software v4.1, Aperio ScanScope XT and Aperio AT2 Console ver. 102.0.7.5
Data analysis	FlowJo 10, GraphPad Prism v9.5.0, Microsoft PowerPoint 365 v2308, Microsoft Excel 365 v2208, ImageScope v12.4 w/ PPC algorithm v9.1, QuPath v0.4.1

For manuscripts utilizing custom algorithms or software that are central to the research but not yet described in published literature, software must be made available to editors and reviewers. We strongly encourage code deposition in a community repository (e.g. GitHub). See the Nature Portfolio guidelines for submitting code & software for further information.

Data

Policy information about availability of data

All manuscripts must include a data availability statement. This statement should provide the following information, where applicable:

- Accession codes, unique identifiers, or web links for publicly available datasets
- A description of any restrictions on data availability
- For clinical datasets or third party data, please ensure that the statement adheres to our policy

Data supporting this work are available within this manuscript and Supplementary Information; source data are provided with this paper.

Research involving human participants, their data, or biological material

Policy information about studies with human participants or human data. See also policy information about sex, gender (identity/presentation), and sexual orientation and race, ethnicity and racism.

Reporting on sex and gender	N/A
Reporting on race, ethnicity, or other socially relevant groupings	N/A
Population characteristics	N/A
Recruitment	N/A
Ethics oversight	N/A

Note that full information on the approval of the study protocol must also be provided in the manuscript.

Field-specific reporting

Please select the one below that is the best fit for your research. If you are not sure, read the appropriate sections before making your selection.

☒ Life sciences ☐ Behavioural & social sciences ☐ Ecological, evolutionary & environmental sciences

For a reference copy of the document with all sections, see nature.com/documents/nr-reporting-summary-flat.pdf

Life sciences study design

All studies must disclose on these points even when the disclosure is negative.

Sample size	No sample size calculation was used. For most conditions, we used 5 bats per cohort, with two cohorts having 4 bats. A total of 33 juvenile bats were used in this study. This was necessary given the limits of our BSL4-based animal workflow and the limited number of available ERBs. Previous work with these bats (Schuh, et al., Nat Comms, 2017; Schuh et al, Scientific Reports, 2022; Jones et al., Viruses, 2019; Guito et al., Current Biology, 2021) has shown that we can achieve statistically significant data from groups of this size.
Data exclusions	No data was excluded from the analysis.
Replication	Due to the logistics, costs and ethical considerations to reduce and refine animal work, experimental replicates were not possible. However, to incorporate biological variation, we used multiple bats per group.
Randomization	Allocation into groups was performed randomly among a pool of juvenile ERBs (8-10 month old) that were available for experimentation, with individual bats tagged with random RFID identification numbers and divided into groups at random. Each group also contained a random number of males and females; previous data from experimentally infected and wild-caught infected bats has shown that sex does not appear to have any impact on Marburg virus infections, virus replication, pathogenesis, etc.
Blinding	Investigators were blinded to group allocations during flow cytometry analysis, as well as histology and immunohistochemistry analyses and quantifications, for which samples were randomly numbered.

Reporting for specific materials, systems and methods

We require information from authors about some types of materials, experimental systems and methods used in many studies. Here, indicate whether each material, system or method listed is relevant to your study. If you are not sure if a list item applies to your research, read the appropriate section before selecting a response.

Materials & experimental systems

n/a	Involved in the study	
☐	☒	Antibodies
☒	☐	Eukaryotic cell lines
☒	☐	Palaeontology and archaeology
☐	☒	Animals and other organisms
☒	☐	Clinical data
☒	☐	Dual use research of concern
☒	☐	Plants

Methods

n/a	Involved in the study	
☒	☐	ChIP-seq
☐	☒	Flow cytometry
☒	☐	MRI-based neuroimaging

Antibodies

Antibodies used	Anti-CD11b-APC/Fire 750 (BioLegend, clone ICRF44, Cat #301302), ERB-specific anti-CD14-PE and anti-CD19-APC (custom designed as described in https://doi.org/10.1016/j.cub.2020.10.015 [Guito et. al, 2021]), and anti-MHCII-BV786 (BD Biosciences, clone 2G9, Cat #743875), mouse monoclonal anti-CD3 (Dako/Agilent Technologies, clone F7.2.38, code number M7254), mouse monoclonal anti-CD79a (Biocare Medical, clone HM47/A9, code number CM067), rabbit polyclonal anti-ionized calcium-binding adapter molecule 1 (Iba1, Wako, Cat #019-19741) and rabbit monoclonal anti-Ki-67 (Cell Marque Corporation, clone SP6, code number 275R).
Validation	ERB-specific CD14-PE and CD19-APC were validated as described in https://doi.org/10.1016/j.cub.2020.10.015 (Guito et. al, 2021). Validations for commercial antibodies described above can be found at: CD11b (https://www.biolegend.com/de-de/products/purified-anti-human-cd11b-antibody-770), MHCII (https://www.bdbiosciences.com/en-us/products/reagents/flow-cytometry-reagents/research-reagents/single-color-antibodies-ruo/bv786-rat-anti-mouse-i-a-i-e.743875), CD3 (https://www.agilent.com/en/product/immunohistochemistry/antibodies-controls/primary-antibodies/cd3-%28concentrate%29-76649#productdetails), CD79a (https://biocare.net/product/cd79a-antibody/), Iba1 (https://labchem-wako.fujifilm.com/us/product/detail/W01W0101-1974.html), Ki-67 (https://www.cellmarque.com/antibodies/CM/108/Ki-67_SP6).

Animals and other research organisms

Policy information about studies involving animals; ARRIVE guidelines recommended for reporting animal research, and Sex and Gender in Research

Laboratory animals	Rousettus aegyptiacus (Egyptian rousette bats), 8-10 mo old, captive-bred.
Wild animals	This study did not involve wild animals.
Reporting on sex	Each bat group contained a random number of males and females. Sex was assigned by inspecting the external genitalia. Out of the 33 juvenile bats in the study, 20 were male and 13 were female. Given our limitations for group numbers, as well as previous data from experimentally infected and wild-caught infected bats, sex does not appear to have any detectable impact on infections, virus replication, pathogenesis, etc., therefore we did not perform sex-based analysis in this study.
Field-collected samples	This study did not involve samples collected from the field.
Ethics oversight	The study with Egyptian rousette bats described in this manuscript was performed in accordance with approved animal protocols (#2977, #3090), policies and practices overseen by CDC's Institutional Animal Care & Use Committee (IACUC), Animal Care and Use Program Office (ACUPO), Institutional Biosecurity Board (IBB), and Comparative Medicine Branch (CMB).

Note that full information on the approval of the study protocol must also be provided in the manuscript.

Flow Cytometry

Plots

Confirm that:

- [x] The axis labels state the marker and fluorochrome used (e.g. CD4-FITC).
- [x] The axis scales are clearly visible. Include numbers along axes only for bottom left plot of group (a 'group' is an analysis of identical markers).
- [x] All plots are contour plots with outliers or pseudocolor plots.
- [x] A numerical value for number of cells or percentage (with statistics) is provided.

Methodology

Sample preparation	Total splenocytes and axillary lymph node (ALN) cells from Marburg virus infected Egyptian rousette bats were isolated and prepared as described in https://doi.org/10.1016/j.cub.2020.10.015 (Guito et. al, 2021) using the Spleen Dissociation Kit (mouse) enzyme mix (Miltenyi) and run on a gentleMACS Octo Dissociator with Heaters (program 37C_m_SDK_1). RPMI supplemented with 10% FBS and Benzonase were added to cell suspensions and passed through 70 µm strainers into conical tubes. Pellets were resuspended in ACK Lysing Buffer and incubated at room temperature, washed and resuspended in RPMI/Benzonase, then enumerated using an ORFLO MOXI Z Mini cell counter. Cells were then stained with a fluorescently-conjugated antibody cocktail and fixed using BD CytoFix prior to running on the cytometer.
Instrument	Stratedigm S1400EXi cytometer platform
Software	Stratedigm CellCapTure software ver. 4.1 for data collection, and then FlowJo 10 (TreeStar) for data analysis.
Cell population abundance	We analyzed the viability and percentage of highly abundant cell types (lymphocytes and myeloid cells) from spleen and lymph node preparations.
Gating strategy	Data analyzed blindly was gated first by removing debris, then selecting for singlets. Cell viability (splenocytes and ALN cells) was then assessed by gating to exclude positive live/dead-stained cells, compared to total cells with forward scatter (FSC) and side scatter (SSC) characteristics of total leukocytes. Percent of viable cells was gated by comparing live cells verses total

leukocytes and was used to calculate the total viable cells per tissue using the absolute cell counts from the dissociation of splenocytes/LN cells. Splenic CD11b+ and CD14+ cells were then determined as a percentage of total viable cells, after gating on live cells and total leukocytes. Splenic B cells (CD19+) were determined by gating on live cells, then cells with FSC/SSC characteristics typical of lymphocytes, and finally the CD19+ proportion. Splenic T cells (CD19-) were estimated by calculating the remaining cells in the lymphocyte gate after gating out the CD19+ population, which likely also include other lymphocyte subtypes (bat NK and NKT cells).

☒ Tick this box to confirm that a figure exemplifying the gating strategy is provided in the Supplementary Information.
